# Genetic diversity in L1 ORF of human papillomavirus in women with cervical cancer with and without human immunodeficiency virus in Botswana and Kenya

**DOI:** 10.1186/s12879-022-07081-3

**Published:** 2022-01-27

**Authors:** Leabaneng Tawe, Wonderful T. Choga, Giacomo M. Paganotti, Ontlametse T. Bareng, Tlhalefo D. Ntereke, Pleasure Ramatlho, Doreen Ditshwanelo, Simani Gaseitsiwe, Ishmael Kasvosve, Doreen Ramogola-Masire, Omenge E. Orang’o, Erle Robertson, Nicola Zetola, Sikhulile Moyo, Surbhi Grover, Aaron C. Ermel

**Affiliations:** 1 Department of Medical Laboratory Sciences, Faculty of Health Sciences, University of Botswana, Private Bag 00712, Gaborone, Botswana; 2 Botswana-University of Pennsylvania Partnership, Gaborone, Botswana; 3 Botswana Harvard AIDS Institute Partnership, Gaborone, Botswana; 4 Division of Human Genetics, Faculty of Health Sciences, University of Cape Town, Cape Town, South Africa; 5 Division of Infectious Diseases, Perelman School of Medicine, University of Pennsylvania, Philadelphia, PA USA; 6 Department of Biomedical Sciences, Faculty of Medicine, University of Botswana, Gaborone, Botswana; 7 Department of Immunology and Infectious Diseases, Harvard School of Public Health, Boston, MA USA; 8 Department of Obstetrics and Gynaecology, Faculty of Medicine, University of Botswana, Gaborone, Botswana; 9 Moi University, Eldoret, Kenya; 10 Department of Otorhinolaryngology, Perelman School of Medicine, University of Pennsylvania, Philadelphia, PA USA; 11 Department of Radiation Oncology, Perelman School of Medicine, University of Pennsylvania, Philadelphia, PA USA; 12 Indiana University School of Medicine, Indianapolis, IN USA

**Keywords:** Botswana, Kenya, Cervical cancer, Human papillomavirus, HPV variants phylogenetic analysis, HIV, *L1* gene, HIV co-infection

## Abstract

**Background:**

The variation of human papillomavirus (HPV) genotypes shapes the risks of cervical cancer and these variations are not well defined in Africa. Nucleotide changes within the *L1* gene, nucleotide variability, and phylogeny were explored in relation to HIV in samples from Botswana and Kenya.

**Methods:**

A total of 98 HPV-positive cervical samples were sequenced to identify different HPV variants. Phylogenetic inferences were used to determine HPV genotypes and investigate the clustering of sequences between women living with HIV (WLWHIV) and -women not living with HIV (WNLWHIV).

**Results:**

Out of 98 generated sequences, 83.7% (82/98) participants had high-risk (HR) HPV genotypes while 16.3% (16/98) had low-risk (LR) HPV genotypes. Among participants with HR-HPV genotypes, 47.6% (39/82) were coinfected with HIV. The prevalence of HR-HPV genotypes was statistically higher in the Botswana population compared to Kenya (p-value < 0.001). Multiple amino acid mutations were identified in both countries. Genetic diversity differed considerably among WLWHIV and WNLWHIV. The mean pairwise distances between HPV-16 between HIV and HIV/HPV as well as for HPV-18 were statistically significant. Six (6) new deleterious mutations were identified in the HPV genotypes based on the sequencing of the L1 region, HPV-16 (L441P, S343P), HPV-18 (S424P), HPV-45 (Q366H, Y365F), and HPV-84 (F458L). The majority of the patients with these mutations were co-infected with HIV.

**Conclusions:**

Genomic diversity and different genomic variants of HPV sequences were demonstrated. Candidate novel mutations within the *L1* gene were identified in both countries which can be further investigated using functional assays.

**Supplementary Information:**

The online version contains supplementary material available at 10.1186/s12879-022-07081-3.

## Background

Cervical cancer continues to be a major public health problem particularly in less-resourced countries with an estimated 570,000 cases diagnosed, and 311,000 death rates recorded in 2018 [1]. In sub-Saharan Africa, cervical cancer is the most common cancer and its accounts for 22.2% of all the cancers in women and it is the also the most common cause of cancer death among women [2]. It is one of the most common cancers in women living with human immunodeficiency virus (HIV) and presents a significant public health threat to women especially on the African continent. In sub-Saharan Africa, human papillomavirus (HPV)-associated cervical cancer is an important cause of morbidity and mortality [3]. Cervical cancer has been recognized as a rare outcome of a sexually transmitted infection, and the etiology is limited to a few HPV genotypes. HPV infections can be facilitated by co-infection with HIV [4]. To date, over 200 HPV genotypes have been identified and classified according to their mucosal types, they are divided into high risk (HR), possible or probable high risk and low risk (LR) HPV genotypes, depending on their association with the development of cancer. For decades, 14 highly carcinogenic HPV genotypes (-16, -18, -31, -33, -35, -39, -45, -51, -52, -56, -58, -59, -66 and -68) have been recognized as the causative agent of cervical cancer [5, 6]. However, HPV genotypes-16 and -18 have been demonstrated and identified as the predominant oncogenic genotypes of all cervical cancer cases worldwide [7]. Despite having the highest burden of risk factors associated with HPV infection, persistence, and progression to cervical cancer [8], comprehensive data on HPV genotypes and profiles of HPV mutations associated with different cervical cancer risk groups such as HIV/HPV co-infected in Africa are lacking.

Molecular epidemiological studies assessing the phylogenetic association of HPVs based on oncogenic risk and supporting specific biological and pathological traits distinctive to HPV genera, species and types are still limited. Previous studies in Africa have explored the frequency and distribution of the HPV genotypes in HIV-infected women [9], with consistent results about the influence of HIV on HPV genotype distribution. Several studies in Africa have also explored the genomic diversity of HPV variants [10–15]. HPV genotypes-16, -18, -45, and -58 were observed among most cervical samples studied from Botswana and Kenya [3, 14, 16–26].

The objective of this study was to use Sanger genotyping protocol to detect and genotype HPV isolates in cervical cancer specimens of patients’ living- and not living- with HIV obtained from Botswana and Kenya. Apart from determining the prevalence and distribution of HPV genotypes (LR and HR) among the two groups (HPV versus HIV/HPV-coinfected), the mutation profiles (nucleotide) and amino acid changes) within *L1* region of HPV genotypes were used to determine the mean pairwise distances and assessing for any signature mutations associated with cervical cancer among the HIV/HPV-coinfected patients. Phylogenetic analyses were used to (i) assign HPV genotypes, and (ii) determine clustering of sequences within and between the two countries.

## Methods

### Study design and population

Formalin-fixed paraffin-embedded (FFPE) tissue specimens from women living with HIV (WLWHIV) and -women not living with HIV (WNLWHIV), diagnosed with invasive cervical cancer previously typed using the Abbot Real-Time polymerase chain reaction (PCR) and Linear Array HPV Genotyping Test, LA-HPV (Roche Applied Sciences, Indianapolis, IN) from prior retrospective cross-sectional studies [19, 21] from Botswana and Kenya were utilized.

We proposed to describe the sequence variation of samples with single HPV infections for the two HR-HPV genotypes (HPV-16 and -18) which are responsible for the majority of cervical cancer cases reported in both countries within the ~ 450 base pairs (bp) region amplified by these HPV genotyping systems. Only samples harbouring HPV-16 or -18 were used in this study, samples with mixed HPV infection were excluded from this study. Demographic and clinical data including age, cancer stage, country of origin and HIV status were obtained for samples from Botswana. However, only HIV status was available for Kenya samples. Cervical cancer staging was performed as previously described [21].

### DNA extraction

DNA extraction was performed from the tissue samples archived using a previously established protocol [27]. The extracted DNA was stored at − 80 °C prior to analysis. For the samples from Botswana, the presence of HR-HPV DNA was detected using Abbot Real-Time PCR (Abbot Molecular Inc., Chicago) [21]. While specimens from Kenyan women, HR-HPV detection and genotyping had been previously performed using the LA-HPV [19].

### Polymerase chain reaction (PCR) and sequencing

To isolate a region of *L1* targeted by the LA-HPV and the Abbott Real-Time PCR method, conventional PCR for HPV DNA viral amplification of a ~ 450 bp HPV-specific segment from the *L1* gene covering nucleotide positions (5722-6162) numbered according to NC001526 HPV-16 reference genome was performed using the forward primer MY09 5′-CGTCCMARRGGAWACTGATC-3′ and the reverse primer MY11 5′-GCMCAGGGWCATAAYAATGG-3′. Five microliters of DNA were added to 15 μL of reaction mix containing 1 × PCR buffer, 0.2 mM dNTPs, 4 mM MgCl_2_, 0.3 μΜ of each primer, and 2 U/μL of Platinum Taq DNA Polymerase, High fidelity (Invitrogen, USA).

The thermocycling conditions were denaturing at 96 °C for 10 s, annealing at 50 °C for 5 s, and final extension at 60 °C for 4 min for 25 cycles. PCR products were subjected to electrophoresis in 4% agarose (Applichem) using 1 × TBE buffer (Applichem) and visualized under UV light. PCR products were then purified using commercially available SureClean Plus (Bioline) and the purified products were sequenced directly via automated sequencing using two overlapping PCR primers (both forward and reverse). The BigDye Terminator v3.0 kit (Applied Biosystems; Foster City, CA, USA) was used for sequencing using the automated Sequencer (ABI PRISM 3130xl; Applied Biosystems).

### Sequencing and sequence editing

**Fig. 1:**
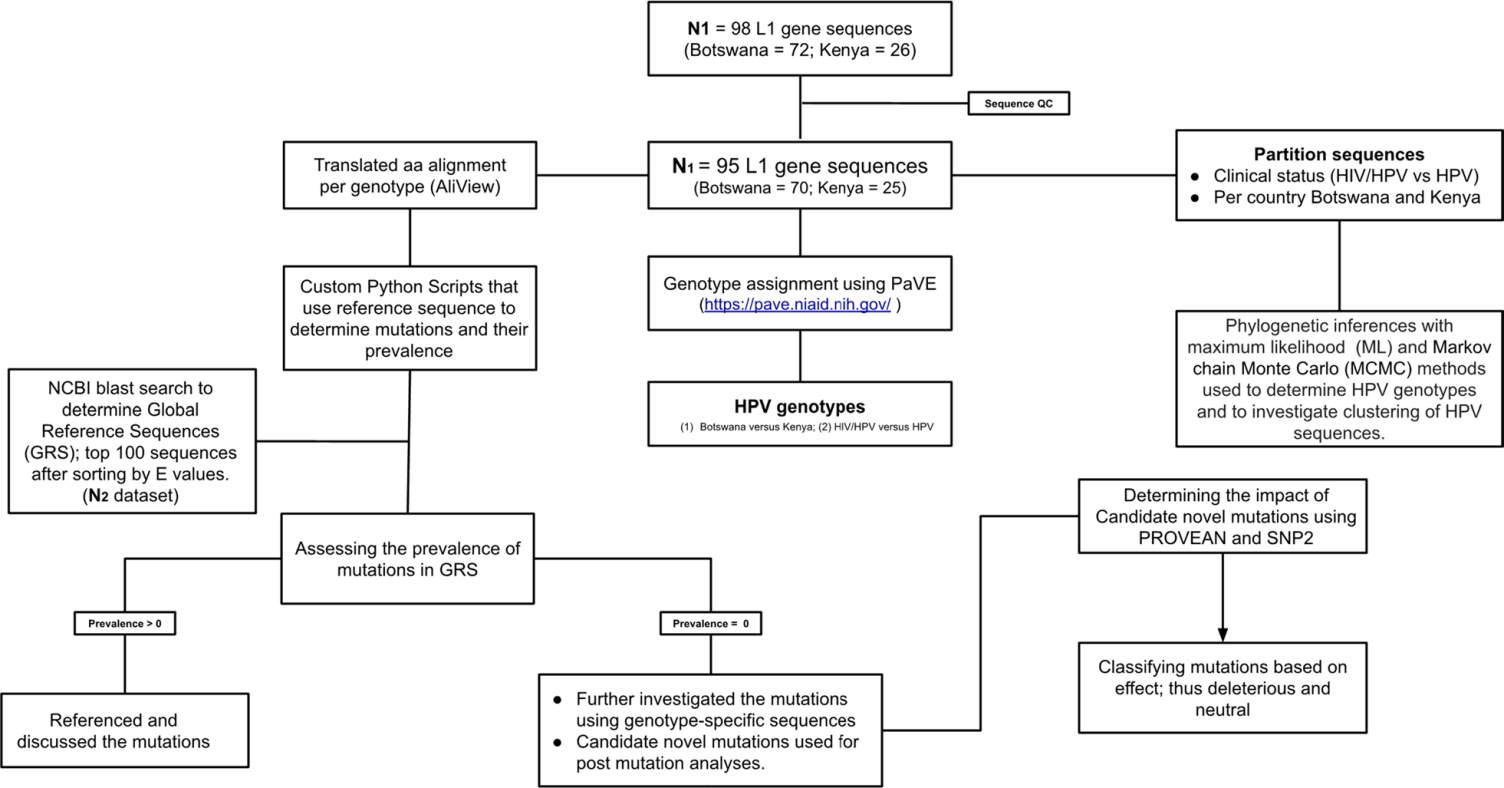
Scheme used to achieve study objectives

The generated chromatographs were quality assessed using Sequencher v5.0 software (Gene Codes Corp., Ann Arbor, MI, USA) followed by generation of consensus sequences in FASTA file format, which were used, for downstream analysis. An online *L1* Taxonomy Tool Analysis Results was utilized to check for similarity with the known HPV reference sequences. Additionally, the BLAST tool was used to determine the subset of sequences with high E^O^ value that can be included in the phylogenetic tree. Finally, referenced alignments per each genotype were performed using AliView version 1.26 and were used for phylogenetic analyses while the translated alignment was used for mutation analysis. We developed a specific workflow to answer the study objectives (Fig. 1).

### Phylogenetic analysis

Phylogenetic inference was performed using the Bayesian Markov chain Monte Carlo (MCMC) approach implemented in the Bayesian Evolutionary Analysis with the Sampling Trees software (BEAST v1.10.2) with an uncorrelated log-normal relaxed molecular clock, General Time Reversible substitution model, and gamma site heterogeneity. The MCMC was set at a chain length of 500,000,000 with parameters logged every 10,000. The tree was visualized in FigTree v1.4.3 after a 10% burn-in using Tree Annotator v1.8.4. Posterior probabilities 0.90 and above were noted as statistically significant. The sequences generated in this study are available in GenBank under accession numbers (awaiting accession numbers).

### HPV-specific mutation analysis

To assess for any known mutations or signatures amino acids that may be associated with immune pressure among HPV patients in Botswana and Kenya, 95 aligned sequences with high coverage were included in the analysis (N_1_ dataset). Here, the top 100 sequences after sorting by E value and covering the full *L1* region were obtained from the NCBI blast search and compared to the N_1_ dataset. Comparisons were done at the *aa* level to exclude synonymous polymorphisms. Genetic diversity and any signature mutations associated with HIV/HPV co-infection were investigated by comparing the HPV sequences isolated from patients with HPV mono-infection versus those with without. Mutations that had not been noted previously in the literature were termed “candidate novel mutations”. The candidate novel mutations were further assessed using Protein Variation Effect Analyzer (PROVEAN) [28] and SNAP2 [29] to determine their impact at the gene level. Briefly, PROVEAN classifies mutations with a negative impact on protein biological function as deleterious and SNAP2 uses ‘effect’ or ‘neutral’ to indicate the presence or absence of change in protein function caused by a mutation.

### Statistical analysis

Raw data were collected, processed and coded using Excel. Study demographics were presented as percentages for categorical variables and compared among participants with HR-HPV and LR-HPV HPV genotype using the Chi-square test. Wilcoxon rank-sum test was utilised to compare continuous variables. Differences among the prevalence of HPV genotypes in Botswana and Kenya was assessed using a comparison of proportion test. All the statistical analysis was done using Stata version 15 (Stata Corp, College Station, TX, USA) and p-value < 0.05 was considered as statistically significant.

## Results

### Population characteristics

**Fig. 2:**
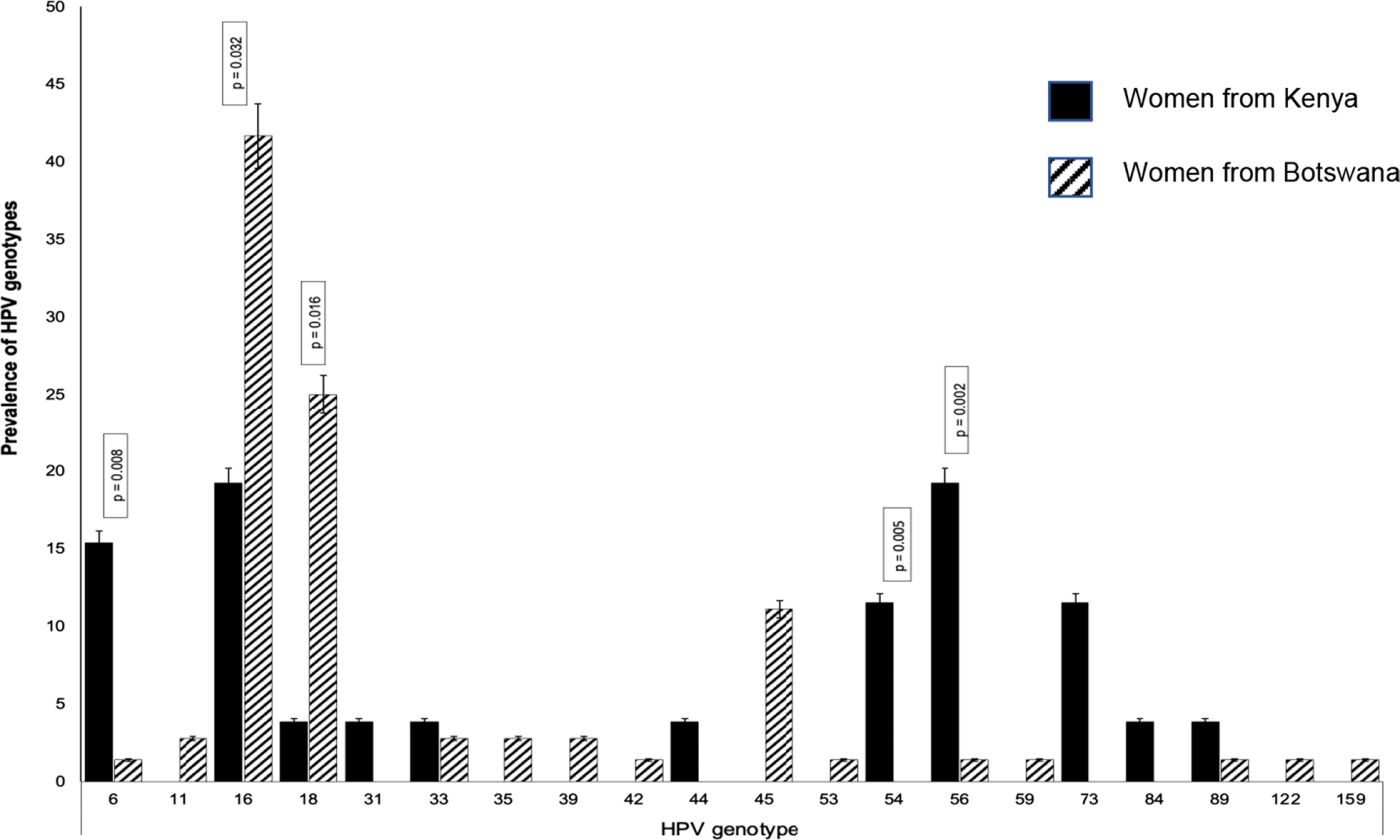
HPV genotypes among women with cervical cancer from Botswana and Kenya

The present study analysed 98 samples positive for HPV-16 or -18 only from 2 previous studies [19, 21]. All samples with mixed HPV infection were excluded from the study. Study participants were a subpopulation of samples from Botswana (n = 72) and another from Kenya (n = 26). Baseline demographics and clinical characteristics of the study participants are summarised in Table 1. The distribution of HPV genotypes stratified by country are shown in Fig. 2. Out of 98 generated sequences, 83.7% (82/98) participants had HR-HPV genotypes while 16.3% (16/98) had LR-HPV genotypes (Table 1). However, in some patients, we identified other additional HPV genotypes which were previously missed when the same samples were genotyped using Abbott and LA-HPV. Among participants with HR-HPV genotypes, 51.2% (42/82) were coinfected with HIV (HIV/HPV), while 47.5% (39/82) were from Botswana and 3.7% (3/82) were from Kenya based on MY09/11 Sanger sequencing-based method. The most predominant cancer stage among participants from Botswana was stage 2. Amongst individuals with LR-HPV genotypes, 12.5% were infected with HIV, cancer stage 3 was the most predominant cancer stage. The prevalence of HR-HPV genotypes was significantly higher in the Botswana population compared to Kenya (p-value < 0.001). We did not record any statistical significance in other variables among HR and LR-HPV genotypes among the Botswana population.

**Table 1:** Baseline demographics and clinical characteristics for participants

	Total n = 98	HR-HPV n = 82	LR-HPV n = 16	P-value
Median Age in years Median (IQR)	50 (42–54)	50 (42–61)	51.5 (47.5–57)	0.67^a^
Country, n (%)				
Botswana	72 (73.5)	66 (80.5)	6 (37.5)	< 0.001^b^
Kenya	26 (26.5)	16 (19.5)	10 (62.5)	
HIV status, n (%)	n = 72	n = 66	n = 6	
HIV negative	31 (43.1)	27 (40.9)	4 (66.7)	
HIV positive	41 (56.9)	39 (59.1)	2 (33.3)	0.22^b^
Cancer stage, n (%)**	n = 43	n = 39	n = 4	
Stage 1	6 (14.0)	6 (15.4)	0 (0.0)	0.69^b^
Stage 2	21 (48.8)	18(46.2)	3 (75.0)	
Stage 3	15 (34.9)	14 (35.9)	1 (25.0)	
Stage 4	1 (2.3)	1 (2.6)	0 (0.0)	

*HIV* human immunodeficiency virus, *HPV* human papillomavirus, *HR* high risk, *LR* low risk, *IQR* interquartile range

^a^P-value was calculated using the Rank sum test

^b^P-values were obtained by chi-square test

**Cancer stage was only available for Botswana data

### HPV genotypes

**Fig. 3:**
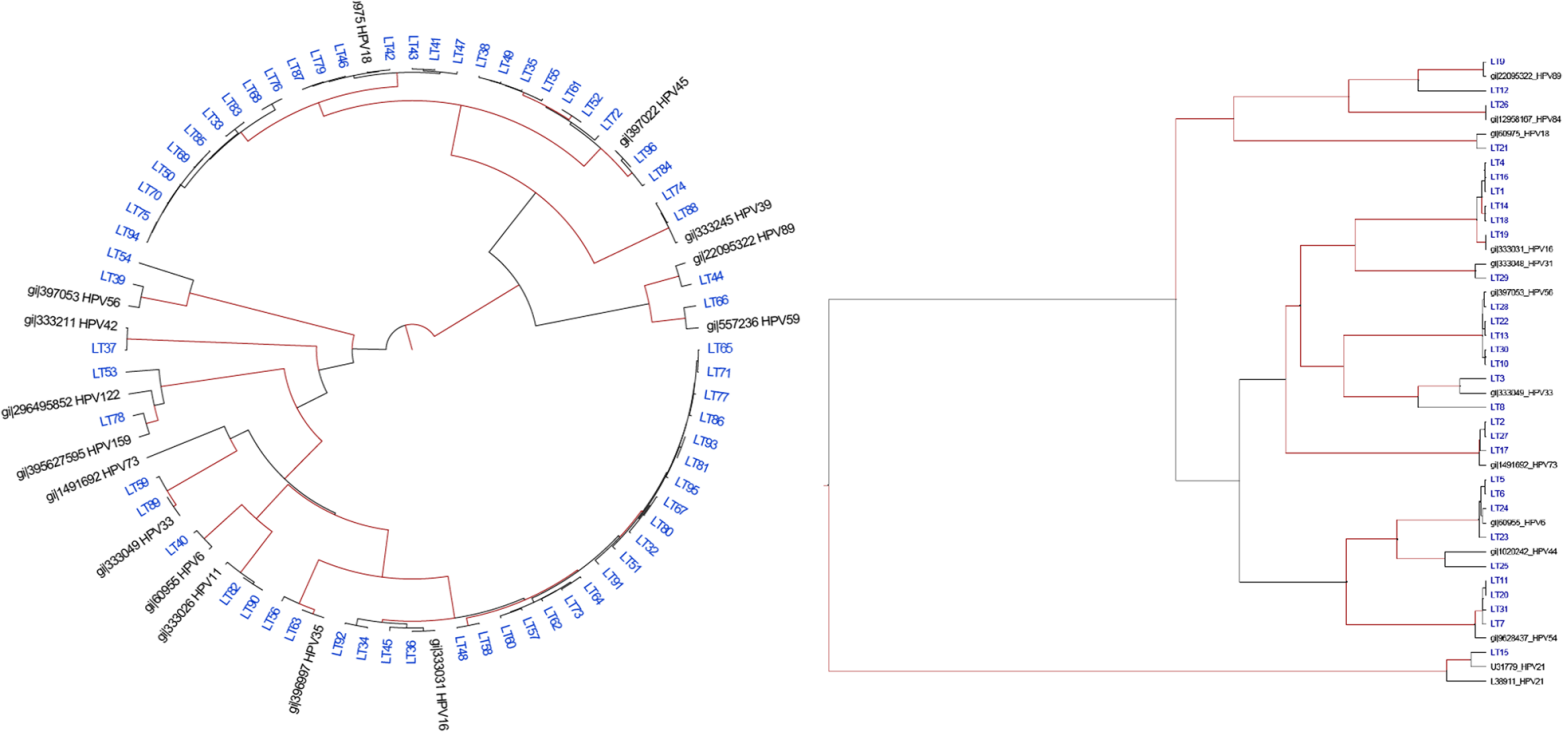
A phylogenetic tree of the *L1* region (~ 450 bp) covering nucleotide positions (5722-6162) numbered to NC001526 HPV-16 reference genome) used to assign HPV types to sequences in this study. Trees were constructed using BEAST method. Strains from Botswana sequenced in the present study are shown in the tree (left), while Kenya sequences are shown in the tree (right). Reference strains are designated by their accession number. All positions with less than 95% site coverage were eliminated, i.e., fewer than 5% alignment gaps, missing data, and ambiguous bases were allowed at any position (partial deletion option). There were a total of 339 positions in the final dataset. Evolutionary analyses were conducted in BEAST v1.2

Human papillomavirus genotypes were identified using both phylogenetic tree (Fig. 3) and online *L1* Taxonomy Tool Analysis. After quality control (QC), 3 sequences were excluded for mutation and phylogenetic analyses because sequences were too short (< 450 bp). In total, 19 HPV genotypes were determined, -6, -11, -16, -18, -35, -39, -42, -45, -53, -54, -55, -56, -58, -59, -73, -81, -89, -122, and -159 (Fig. 2). Out of 19 different genotypes found in Botswana and Kenya populations, five showed statistically significant differences in their frequency (p-value < 0.05) between countries (Fig. 2). Thus, HPV genotypes-6, -54 and -73 were higher in Kenyan population while genotypes-16 and -18 were recorded higher in Botswana population. The WLWHIV had multiple HPV genotypes compared to WNLWHIV even though the difference was not statistically significant (p-value > 0.05).

### Phylogenetic analysis

**Fig. 4:**
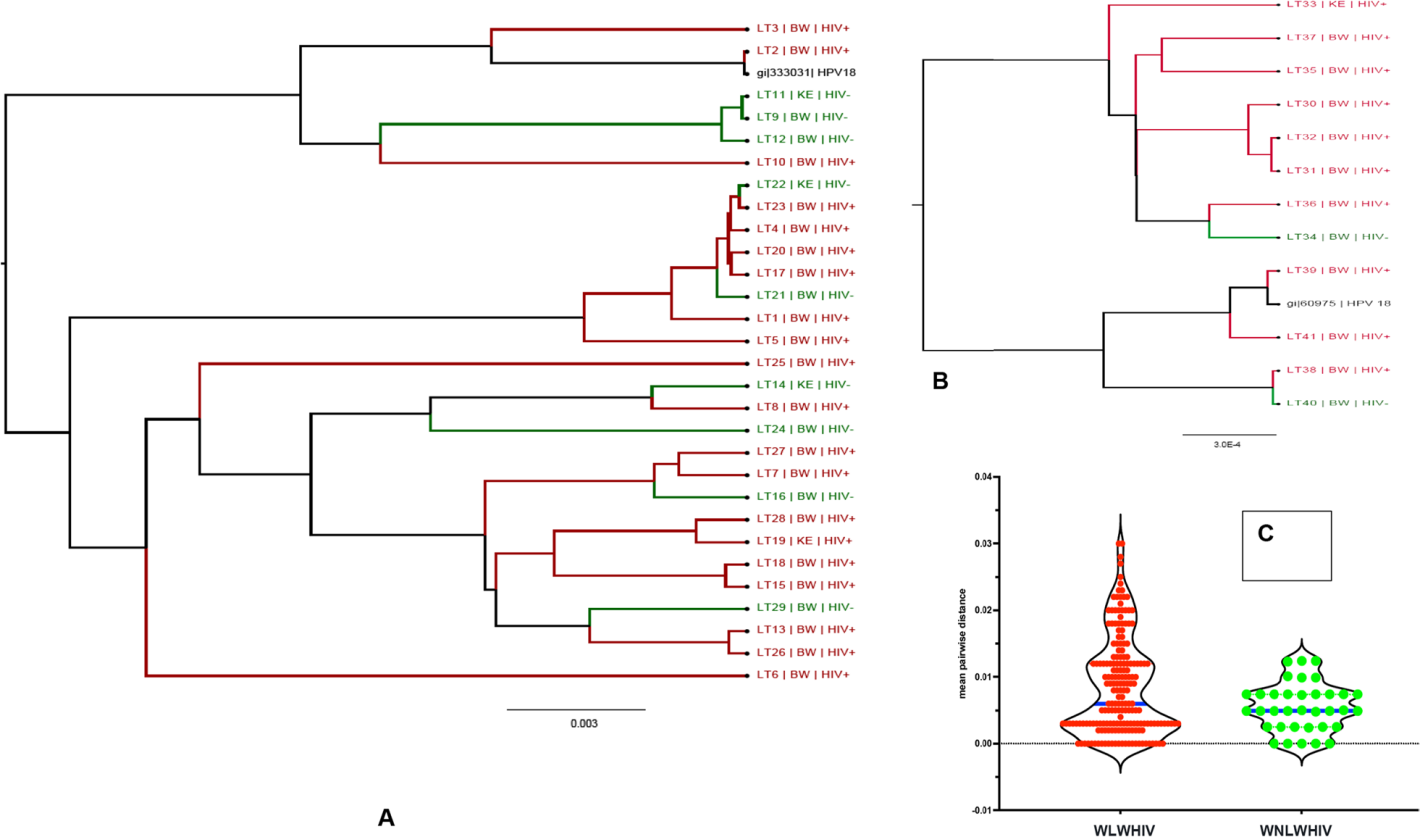
Phylogenetic analysis using BEAST tree for HPV-16 (**A**) and -18 (**B**) sequences from the present study. Mean pairwise distribution of HPV 16 sequences isolated from WLWHIV versus WNLWHIV (**C**)

Phylogenetic analysis included all the 95 HPV sequences obtained in this study that were adequate for phylogeny (> 400 bp sequence length). All the studied HPV sequences clustered with reference and had posterior probabilities > 90%, and could be used to assign the genotypes with confidence. Phylogenetic inference with maximum likelihood and MCMC methods showed that there were no isolated clusters among HPV sequences from both countries. This was also true when trees for HPV-16 (n = 29) and HPV-18 (n = 12) sequences from this study and the respective GenBank references for the *L1* gene region were constructed (Fig. 4). However, there was a general increase in nucleotide genetic diversity among the HPV sequences isolated from WLWHIH as opposed to WNLWHIV (Fig. 4) as shown by multiple branches within the phylogenetic tree. The overall mean pairwise distances of HPV-16 and HPV-18 sequences isolated from WLWHIV versus WNLWHIV were not statistically significant.

### Mutations analysis

#### Nucleotide diversity

In total, 68 nucleotide base substitutions were detected using reference sequences for each genotype. Additional file 1: Table S1 summarises the different polymorphisms detected per genotype and only genotypes with counts greater than 1 per strata were considered. Although there were more HPV types among WLWHIV, most of the HPV genotypes among WLWHIV were HR-HPV genotypes.

#### Mutations at the amino acid level

The present study defined mutations as amino acid changes that differed from that of reference sequence and candidate escape mutations were those that have not yet been reported in 1000 most similar sequences. Six (6) new variants were identified based on the sequencing of the *L1* region, HPV-16 (L441P, S343P), HPV-18 (S424P), HPV-45 (Q366H, Y365F), belonging to the HPV HR-HPV group and HPV-84 (F458L) belonging to the LR-HPV group.

## Discussion

Given the uniquely large and diverse collection of HPV-genotyped cervical samples worldwide, we were able to evaluate the genetic diversity within HPV genotypes and report on the geographic distribution of HPV variants, as well as measure their association with cervical cancer in Botswana and Kenya. Several studies have investigated HPV prevalence and type-specific distribution in cervical cancer patients from Botswana and Kenya, and found that HPV-16 or -18 (HPV-16/18) were identified in 93.8% from Kenya, and 61.8% from Botswana. Non-HPV-6/18 genotypes were detected in 17.2% from Kenya, and 47.8% from Botswana [18, 19]. Again some studies have shown HPV prevalence in Botswana and Kenya [3, 14, 16–26]. However, the numbers of studies investigating the variants within HPV genotypes are limited. In the present study, the molecular characterization of HPV variants was performed in sequences from Botswana and Kenya. By sequencing the *L1* region of HPV positive cervical samples, we were able to confirm the majority of the previously reported HPV genotypes [14, 16–21, 24–26]. However, other HPV genotypes” were identified through sequencing in samples that were previously positive for HPV-16 or -18 only in both countries. This is likely due to the fact that the MY09/11 primers are universal primers and sequencing is not limited by the presence of HPV type-specific probes as in the LA-HPV and Abbott realtime PCR methods. Again, detection from FFPE samples could have been suboptimal due to nonuniform coverage of the tissue. In some cases, low-level of HPV replication may be missed because of thresholds standards. Overall, the frequency of the genotypes identified in both countries are the same as previously reported, having HPV-16 as the most frequently detected genotype followed by HPV-18 and the rest. The sequencing analysis illustrated that multiple variants were identified in both countries. Variants of HPV that changed the protein sequence of the capsid protein encoded by *L1* were found. These observations, in light of the slow evolution of HPV at the population level, suggest that strong selection pressures are at play in each infection cycle. We analyzed the mutation frequency in the panel for HPV genotypes in WLWHIV and WNLWHIV and found mutations within HPV genotypes. We could not assess the clinical relevance (i.e., association with HIV infection or cancer stage at presentation) of the detected variants due to small sample size. However, it is interesting that the new variations in high-risk genotypes were only identified in samples from HIV patients. The most striking finding in this report is the high proportion of mutations in the HPV‐genotypes (including both those arising among WLWHIV and those arising among WNLWHIV). Amino acid variations were found in the *L1* region of the HPV genotypes (Additional file 1: Table S1). In this study, 6 novel amino acids variations, which were previously unreported in the literature, were found in HPV-16, HPV-18, HPV-45 and HPV-84 (Table 2). However, two of them were found in only one sample (Y365F and F458L) and may have occurred by PCR amplification. These variations could be related to the early promoter activation of HPV and may play a crucial role in the transcriptional modulation of the HPV *L1* oncogenes via the promoter. Variants identified based on the sequencing of the *L1* region are; HPV-16 (L441P, S343P), HPV-18 (S424P), HPV-45 (Q366H, Y365F), belonged to the HPV HR-HPV group and HPV-84 (F458L) for the LR-HPV group. These alterations are described for the first time, and functional implications resulting from this variation need further analyses. Changes in the *L1* region of the HPV genome may be important for discriminating the infectious potential of different variants, as well as in defining epitopes relevant to vaccine design. Some previous studies investigating HPV-16 full-length sequences in cervical specimens have shown that the contiguous deletions identified to be highly associated with cancer are suggestive of a pattern of HPV integration [30–37]. The findings of this study indicate that there could be variants of HPV circulating within sub-Saharan Africa. Further studies are needed to confirm the presence of new HPV variants and genotypes and to understand the evolution of HPV isolates in Botswana and Kenya by analyzing the complete HPV genome or different regions of HPV genes such as *L2*, LCR, *E6* and *E7*.

**Table 2:** Novel genetic mutations found in the 5′ and 3′-ends of HPV-16, -18, -45, -84 -*L1* regions

HPV genotype	Mutations	HIV status	Cancer stage	Count of sequences in GRS	Deleterious
16	L441P	Positive	2	–	√
	S343P	Positive	n/a	–	√
18	S424P	Positive	n/a	–	√
45	Q366H	Positive	3	51	√
84	Y365F	Negative	2	–	√
	F458L	Negative	n/a	–	√

*GRS* gene recruitment sequence, *HIV* human immunodeficiency virus, *HPV* human papillomavirus, *n/a* not applicate

Our work showed genetic variability of *L1*, making it essential to take into account the HPV variants lineages or population stratification when developing vaccines. The limitation of this study was the small sample size for samples from Botswana and Kenya; we could not determine whether the synonymous mutations that are described have any impact on the protein expression. We attempted to investigate HPV full-length sequences in FFPE cervical cancer specimens, but were unable to do so because of highly fragmented samples. Further study is required to determine whether variants represent a higher risk for cervical cancer. Additionally, functional studies regarding HPV polymorphisms across the HPV genome variants should be conducted to explore the biological evidence of carcinogenicity.

## Conclusion

We demonstrated the genomic diversity of HPV sequences and phylogenesis of HPV genotypes in Botswana and Kenya samples giving important information as six new *L1* single amino acids changes were identified. New variations in high-risk genotypes were only identified in samples from HIV patients. However, it was not possible to correlate disease severity with any particular variant. The results illustrated that multiple HPV variants are available in both countries. Further studies with complete sequencing of HPV genomes from large population-based and case–control studies of cervical pre-cancer and cancer are required to understand viral carcinogenesis and possibly to improve preventive and therapeutic strategies in the future.

## Supplementary Information

**Additional file 1: Table S1.** Amino acid sequence variation in sequences of *L1* gene of HPV genotypes amongst cancers from Kenya and Botswana.

## Data Availability

The datasets used and/or analyzed during the current study are available from the corresponding author on reasonable request.

## Abbreviations

HIV: Human immunodeficiency virus
HPV: Human papillomavirus
HR: High risk
LR: Low risk
WLWHIV: Women living with HIV
WNLWHIV: Women not living with HIV
FFPE: Formalin-fixed paraffin-embedded
LA-HPV: Linear array HPV genotyping test
MCMC: Markov chain Monte Carlo
PCR: Polymerase chain reaction
bp: Base pair

## References

[1] Ferlay J, Colombet M, Soerjomataram I, Mathers C, Parkin DM, Piñeros M (2019). Estimating the global cancer incidence and mortality in 2018: GLOBOCAN sources and methods. Int J Cancer.

[2] Parkin DM, Hämmerl L, Ferlay J, Kantelhardt EJ (2020). Cancer in Africa 2018: the role of infections. Int J Cancer.

[3] De Vuyst H, Chung MH, Baussano I, Mugo NR, Tenet V, van Kemenade FJ (2013). Comparison of HPV DNA testing in cervical exfoliated cells and tissue biopsies among HIV-positive women in Kenya. Int J Cancer.

[4] Badial RM, Dias MC, Stuqui B, Melli PPDS, Quintana SM, Bonfim CMD (2018). Detection and genotyping of human papillomavirus (HPV) in HIV-infected women and its relationship with HPV/HIV co-infection. Medicine (Baltimore).

[5] Burd EM (2003). Human papillomavirus and cervical cancer. Clin Microbiol Rev.

[6] Muñoz N, Bosch FX, de Sanjosé S, Herrero R, Castellsagué X, Shah KV (2003). International Agency for Research on Cancer Multicenter Cervical Cancer Study Group. Epidemiologic classification of human papillomavirus types associated with cervical cancer. N Engl J Med.

[7] de Sanjose S, Quint WG, Alemany L, Geraets DT, Klaustermeier JE, Lloveras B, Tous S (2010). Retrospective International Survey and HPV Time Trends Study Group. Human papillomavirus genotype attribution in invasive cervical cancer: a retrospective cross-sectional worldwide study. Lancet Oncol.

[8] Jemal A, Bray F, Center MM, Ferlay J, Ward E, Forman D (2011). Global cancer statistics. CA Cancer J Clin.

[9] Clifford GM, de Vuyst H, Tenet V, Plummer M, Tully S, Franceschi S (2016). Effect of HIV infection on human papillomavirus types causing invasive cervical cancer in Africa. J Acquir Immune Defic Syndr.

[10] Tornesello ML, Buonaguro FM, Meglio A, Buonaguro L, Beth-Giraldo E, Giraldo G (1997). Sequence variations and viral genomic state of human papillomavirus type 16 in penile carcinomas from Ugandan patients. J Gen Virol.

[11] Calleja-Macias IE, Kalantari M, Huh J, Ortiz-Lopez R, Rojas-Martinez A, Gonzalez-Guerrero JF (2004). Genomic diversity of human papillomavirus-16, 18, 31, and 35 isolates in a Mexican population and relationship to European, African, and Native American variants. Virology.

[12] Jendoubi-Ferchichi M, Satouri L, Ghoul F, Malek-Mellouli M, Derbel AM, Makni MK, Reziga H (2018). Phylogeny and classification of human papillomavirus (HPV)16 and HPV18 variants based on E6 and L1 genes in Tunisian women with cervical lesions. Asian Pac J Cancer Prev.

[13] Fitzpatrick MB, Hahn Z, Mandishora RSD, Dao J, Weber J, Huang C (2020). Whole-genome analysis of cervical human papillomavirus type 35 from rural Zimbabwean women. Sci Rep.

[14] Omire A, Budambula NLM, Kirumbi L, Langat H, Kerosi D, Ochieng W (2020). Cervical dysplasia, infection, and phylogeny of human papillomavirus in HIV-infected and HIV-uninfected women at a reproductive health clinic in Nairobi, Kenya. Biomed Res Int.

[15] Pinheiro M, Gage JC, Clifford GM, Demarco M, Cheung LC, Chen Z (2020). Association of HPV35 with cervical carcinogenesis among women of African ancestry: evidence of viral-host interaction with implications for disease intervention. Int J Cancer.

[16] Ramogola-Masire D, McGrath CM, Barnhart KT, Friedman HM, Zetola NM (2011). Subtype distribution of human papillomavirus in HIV-infected women with cervical intraepithelial neoplasia stages 2 and 3 in Botswana. Int J Gynecol Pathol.

[17] Macleod IJ, O'Donnell B, Moyo S, Lockman S, Shapiro RL, Kayembe M (2011). Prevalence of human papillomavirus genotypes and associated cervical squamous intraepithelial lesions in HIV-infected women in Botswana. J Med Virol.

[18] Ermel A, Ramogola-Masire D, Zetola N, Tong Y, Qadadri B, Azar MM (2014). Invasive cervical cancers from women living in the United States or Botswana: differences in human papillomavirus type distribution. Infect Agent Cancer.

[19] Ermel A, Qadadri B, Tong Y, Orang'o O, Macharia B, Ramogola-Masire D, Zetola NM, Brown DR (2016). Invasive cervical cancers in the United States, Botswana and Kenya: HPV type distribution and health policy implications. Infect Agent Cancer.

[20] Rantshabeng P, Kasvosve I, Ndlovu A, Gaseitsiwe S, Moyo S (2019). Prevalence of high-risk human papilloma virus in women with high-grade squamous cell intraepithelial lesions in Botswana using Abbott RealTime HPV assay. PLoS ONE.

[21] Tawe L, MacDuffie E, Narasimhamurthy M, Wang Q, Gaseitsiwe S, Moyo S (2020). Human papillomavirus genotypes in women with invasive cervical cancer with and without human immunodeficiency virus infection in Botswana. Int J Cancer.

[22] Karani LW, Musyoki S, Orina R, Nyamache AK, Khayeka-Wandabwa C, Nyagaka B (2020). Human papillomavirus genotype profiles and cytological grades interlinkages in coinfection with HIV. Pan Afr Med J.

[23] Guthrie BL, Rositch AF, Cooper JA, Farquhar C, Bosire R, Choi R (2020). Human papillomavirus and abnormal cervical lesions among HIV-infected women in HIV-discordant couples from Kenya. Sex Transm Infect.

[24] Menon S, van den Broeck D, Rossi R, Ogbe E, Mabeya H (2017). Multiple HPV infections in female sex workers in Western Kenya: implications for prophylactic vaccines within this sub population. Infect Agent Cancer.

[25] Orang'o EO, Emont JP, Ermel AC, Liu T, Omodi V, Tong Y (2020). Detection of types of HPV among HIV-infected and HIV-uninfected Kenyan women undergoing cryotherapy or loop electrosurgical excision procedure. Int J Gynaecol Obstet.

[26] Luchters SM, Vanden Broeck D, Chersich MF, Nel A, Delva W, Mandaliya K (2010). Association of HIV infection with distribution and viral load of HPV types in Kenya: a survey with 820 female sex workers. BMC Infect Dis.

[27] Tawe L, Grover S, Narasimhamurthy M, Moyo S, Gaseitsiwe S, Kasvosve I (2018). Molecular detection of human papillomavirus (HPV) in highly fragmented DNA from cervical cancer biopsies using double-nested PCR. MethodsX.

[28] Choi Y, Sims GE, Murphy S, Miller JR, Chan AP (2012). Predicting the functional effect of amino acid substitutions and indels. PLoS ONE.

[29] Hecht M, Bromberg Y, Rost B (2015). Better prediction of functional effects for sequence variants. BMC Genomics.

[30] Mane A, Patil L, Limaye S, Nirmalkar A, Kulkarni-Kale U (2020). Characterization of major capsid protein (L1) variants of human papillomavirus type 16 by cervical neoplastic status in Indian women: phylogenetic and functional analysis. J Med Virol.

[31] Ahmed HG, Bensumaidea SH, Alshammari FD, Alenazi FSH, ALmutlaq BA, Alturkstani MZ (2017). Prevalence of human papillomavirus subtypes 16 and 18 among Yemeni patients with cervical cancer. Asian Pac J Cancer Prev.

[32] Ntova CK, Kottaridi C, Chranioti A, Spathis A, Kassanos D, Paraskevaidis E (2012). Genetic variability and phylogeny of high risk HPV type 16, 18, 31, 33 and 45 L1 gene in Greek women. Int J Mol Sci.

[33] Pande S, Jain N, Prusty BK, Bhambhani S, Gupta S, Sharma R (2008). Human papillomavirus type 16 variant analysis of E6, E7, and L1 genes and long control region in biopsy samples from cervical cancer patients in north India. J Clin Microbiol.

[34] Gagnon S, Hankins C, Tremblay C, Forest P, Pourreaux K, Coutlée F, Canadian Women's HIV Study Group (2004). Viral polymorphism in human papillomavirus types 33 and 35 and persistent and transient infection in the genital tract of women. J Infect Dis.

[35] Godi A, Martinelli M, Haque M, Li S, Zhao Q, Xia N (2018). Impact of naturally occurring variation in the human papillomavirus 58 capsid proteins on recognition by type-specific neutralizing antibodies. J Infect Dis.

[36] Mariaggi AA, Péré H, Perrier M, Visseaux B, Collin G, Veyer D (2018). Presence of human papillomavirus (HPV) apolipoprotein B messenger RNA editing, catalytic polypeptide-like 3 (APOBEC)-related minority variants in HPV-16 genomes from anal and cervical samples but not in HPV-52 and HPV-58. J Infect Dis.

[37] Nejo YT, Olaleye DO, Odaibo GN (2019). Molecular characterisation of genital human papillomavirus among women in Southwestern, Nigeria. PLoS ONE.

