# Supplementary material for: Genetic diversity in L1 ORF of human papillomavirus in women with cervical cancer with and without human immunodeficiency virus in Botswana and Kenya
Source: BMC Infect Dis. 2022 Jan 27;22:95. doi: 10.1186/s12879-022-07081-3 (PMC8796425; doi:10.1186/s12879-022-07081-3)
Supplement: Supplementary file 1 — Additional file 1: Table S1. Amino acid sequence variation in sequences of L1 gene of HPV genotypes amongst cancers from Kenya and Botswana. [file 12879_2022_7081_MOESM1_ESM.doc]

**Table S1**: Amino acid sequence variation in sequences of *L1* gene of HPV genotypes amongst cancers from Kenya and Botswana

| **HPV genotype** | **Sequences** | **Mutations [Frequency of Mutation per GRS]** | **Reference** |
| --- | --- | --- | --- |
|  |  |  |  |
| **6** | 5 | N352D, N390D |  |
| **16** | 35 | I391M, G326D **,** C345S, S351L, T353P, T389S, S396P **,** W402P, Q424E, **L441P**, **S343P** | [31-34] |
| **18** | 19 | V323I, **S424P** | [32] |
| **33** | 3 | A424V | [35] |
| **35** | 2 | S348T | [35] |
| **44** | 1 | P349S, S350T, T353N,  E355T, Q356E, A393P |  |
| **45** | 8 | S357G, S357N, E457D, Y365F  **Q366H** | [32] |
| **54** | 3 | V324L, S417P, N350S |  |
| **58** |  | T349N, I386V,D394N, N396D, Q414R, K451R | [32, 36] |
| **59** | 1 | P448T |  |
| **73** | 3 | F370Y, E438D, |  |
| **84** | 1 | Q331E, S348C, A349T, N352S, T353A, E354A, S355A, E356G, P359A, T360S, **Y365F**, D451T, **F458L**, G446D, V369T, V380I, R384Q, V389I,S391A, T395N, D398K, S399A, E403D, V409L, Y422F, A437P, K438A, D441E | [37, 38] |
| **89** | 2 | A353G |  |

GRS, gene recruitment sequence; HPV, human papillomavirus; Bold, novel variants
